# The Magnetorheological Finishing (MRF) of Potassium Dihydrogen Phosphate (KDP) Crystal with Fe_3_O_4_ Nanoparticles

**DOI:** 10.1186/s11671-016-1301-4

**Published:** 2016-02-09

**Authors:** Fang Ji, Min Xu, Chao Wang, Xiaoyuan Li, Wei Gao, Yunfei Zhang, Baorui Wang, Guangping Tang, Xiaobin Yue

**Affiliations:** Shanghai Ultra-Precision Optical Manufacturing Engineering Center, Department of Optical Science and Engineering, Fudan University, Shanghai, 200433 People’s Republic of China; China Academy of Engineering Physics, Institute of Machinery Manufacturing Technology, Mianyang, 621900 Sichuan People’s Republic of China; Laboratory of Precision Manufacturing Technology, China Academy of Engineering Physics, Mianyang, 621900 Sichuan People’s Republic of China

**Keywords:** Fe_3_O_4_, Magnetorheological finishing, KDP

## Abstract

The cubic Fe_3_O_4_ nanoparticles with sharp horns that display the size distribution between 100 and 200 nm are utilized to substitute the magnetic sensitive medium (carbonyl iron powders, CIPs) and abrasives (CeO_2_/diamond) simultaneously which are widely employed in conventional magnetorheological finishing fluid. The removal rate of this novel fluid is extremely low compared with the value of conventional one even though the spot of the former is much bigger. This surprising phenomenon is generated due to the small size and low saturation magnetization (*M*_s_) of Fe_3_O_4_ and corresponding weak shear stress under external magnetic field according to material removal rate model of magnetorheological finishing (MRF). Different from conventional D-shaped finishing spot, the low *M*_s_ also results in a shuttle-like spot because the magnetic controllability is weak and particles in the fringe of spot are loose. The surface texture as well as figure accuracy and PSD1 (power spectrum density) of potassium dihydrogen phosphate (KDP) is greatly improved after MRF, which clearly prove the feasibility of substituting CIP and abrasive with Fe_3_O_4_ in our novel MRF design.

## Background

Potassium dihydrogen phosphate (KDP) is the unique nonlinear single crystal that is large enough as optical frequency conversion and electro-optical switch in high-fluence environment such as inertial confinement fusion (ICF) laser system. However, this crystal presents a great challenge to the ultra-precision manufacturing due to its low hardness, temperature sensitivity, and water solubility [1–3]. Today, the common precise process for KDP is single-point diamond turning (SPDT) [4–6]. Though highly successful of this technique, it would introduce turning grooves and subsurface defects via pure mechanical force which may lower the laser-induced damage threshold (LIDT) in strong laser application. Furthermore, the waviness errors that induced by vibration, straightness, and some other ambient factors are extremely difficult to be eliminated at present [7, 8]. With increased demands on surface quality of KDP ultra-precision manufacturing, more and more novel processing routes are employed to meet the elevated requirements.

Magnetorheological finishing (MRF) is an advanced sub-aperture polishing technology that contains magnetic sensitive carbonyl iron powders (CIPs) and nonmagnetic abrasive incorporated in aqueous medium. The choice of abrasive is directed by the interaction with workpiece such as the physical properties (e.g., hardness) and chemical properties (e.g., chemical durability). It is considered to be an excellent, deterministic process for finishing optics to high precision with few subsurface defects [9–11]. For a typical MRF process, the fluid is pumped and ejected through a nozzle onto a strong magnetic rotating wheel, and the ribbon is stiffened upon passing into the vicinity of workpiece. The removal rate is determined by process parameters as well as material properties of fluid and workpiece [12–14]. The finishing spot in the contacting zone between ribbon and workpiece is characterized to generate removal function arithmetic.

Arrasmith et al. has reported that nonaqueous magnetorheological fluid which was composed of CIPs, nanodiamonds, and dicarboxylic ester could successfully polish a previously diamond-turned KDP to remove all turning grooves without deteriorating the roughness [15]. Menapace et al. and Peng et al. have reported the MRF of KDP via water deliquescence without abrasive which also could achieve the smooth surface [1, 16]. However, the surface quality still does not meet the requirement in engineering and many researches need to be done to obtain better manufacturing level. Firstly, there are at least two kinds of particles (CIPs and abrasives) in the fluid, increasing the difficulty of fluid design and preparation. Secondly, CIPs’ size distribution is 0.5–3 μm and much larger than the value of nanosized abrasives. Due to CIPs inevitably taking part in the material removal during MRF, the existence of huge particles would introduce obviously scratches and block the improvement of finishing quality [17]. Thirdly, Kozhinova et al. showed that soft CIP and silica abrasive with low removal rate might produce better surface roughness in soft ZnS MRF [18]. These motivate us to meliorate the conventional MRF technology via designing a novel finishing fluid with sole, small, and weak magnetic particles and adjustable low removal rate to improve the surface quality of soft KDP finishing.

Fe_3_O_4_ is a common magnetic functional material that has been widely researched in recent years for its outstanding properties and potential applications in various fields, such as ferrofluids, catalysts, colored pigments, microwave absorber, high-density magnetic recording media, and medical diagnostics [19–22]. In addition, the particle’s size can be accurately controlled from nanometer to micrometer with narrow size distribution; it can also be dispersed easily with a surfactant by surface modification due to its chemical activation which is alive [23–25]. In this paper, we put forward an assumption to utilize the Fe_3_O_4_ nanoparticles to substitute conventional CIP and abrasive simultaneously, which means Fe_3_O_4_ plays the role of a magnetic sensitive particle as well as an abrasive. The idea is based on the understanding that small, narrow size distribution, low hardness, and weak magnetic particle with low removal rate are beneficial for the finishing of the ultra-precise surface.

## Methods

The CIPs were purchased from BASF Co., Ltd. (Germany), and Fe_3_O_4_ nanoparticles were obtained from Aipurui Reagent Co. Ltd. (Nanjing, China) without any further surface modification. Other chemical reagents were purchased from Aladdin Chemical Co., Ltd. without purification. The typical magnetorheological fluid consists of basic liquid, functional particles (CIPs and abrasives, or Fe_3_O_4_), and some additives. The dispersion of particles to avoid scratch during finishing was achieved via high-shear emulsification and ultrasonic fragmentation.

The microstructure and morphology were characterization by scanning electron microscopy (SEM, Zeiss Ultra 55) and transmission electron microscopy (TEM, Zeiss Libra 200FE). The magnetic studies were carried out on a Lake Shore 7410 vibrating sample magnetometer (VSM). The finishing spot was taken on a KDP with the ribbon immersion depth of 0.5 mm. The 3D surface morphology of the finished surface was characterized by Taylor Hobson CCI lite with 20× objective of full resolution. After finishing the experiment on a 100 mm × 100 mm KDP via self-developed MRF apparatus and cleaning, the surface figure accuracy was examined and analyzed by Zygo interferometer.

## Results and Discussion

**Fig. 1:**
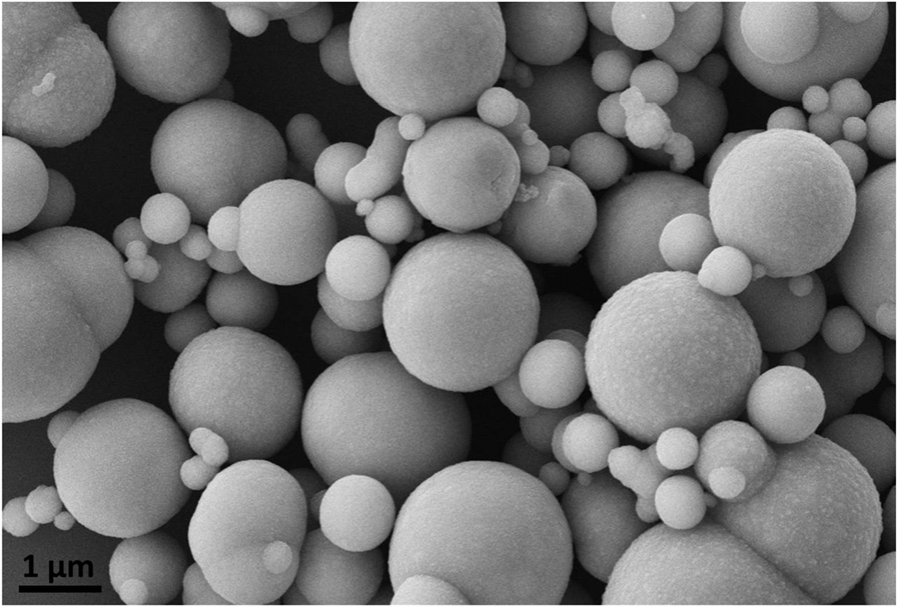
SEM image of CIPs

**Fig. 2:**
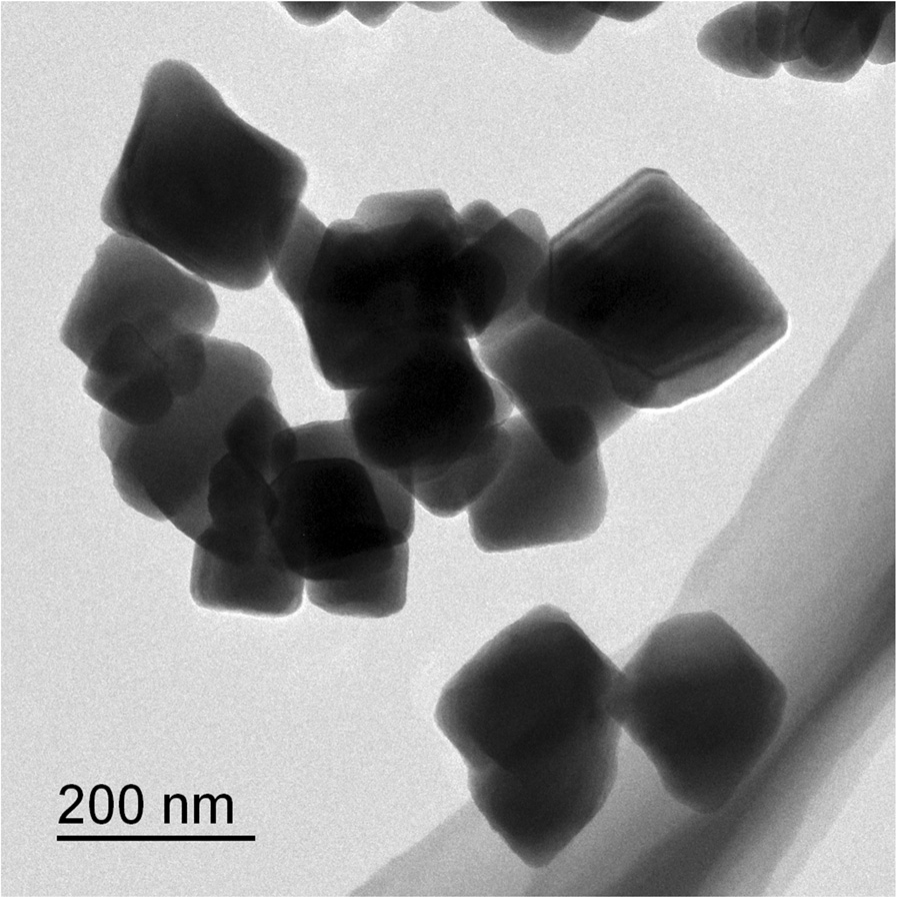
TEM image of Fe_3_O_4_ nanoparticles

Spherical CIP particles with sizes between 0.5–3 μm are shown in Fig. 1; the size distribution is extremely extensive and there are too many large particles. Figure 2 displays the morphology of Fe_3_O_4_ nanoparticles, from which we can see that the size distribution is 100–200 nm with cubic shape, and the edge is clear and four horns of every particle are sharp. Though the particles apparently agglomerate together due to intrinsic static magnetic absorption force, it should be noted that these incompact agglomerations may not greatly influence the subsequently finishing quality because the slurry is stirred online in the delivery system and the soft agglomerations would be dispersed.

**Fig. 3:**
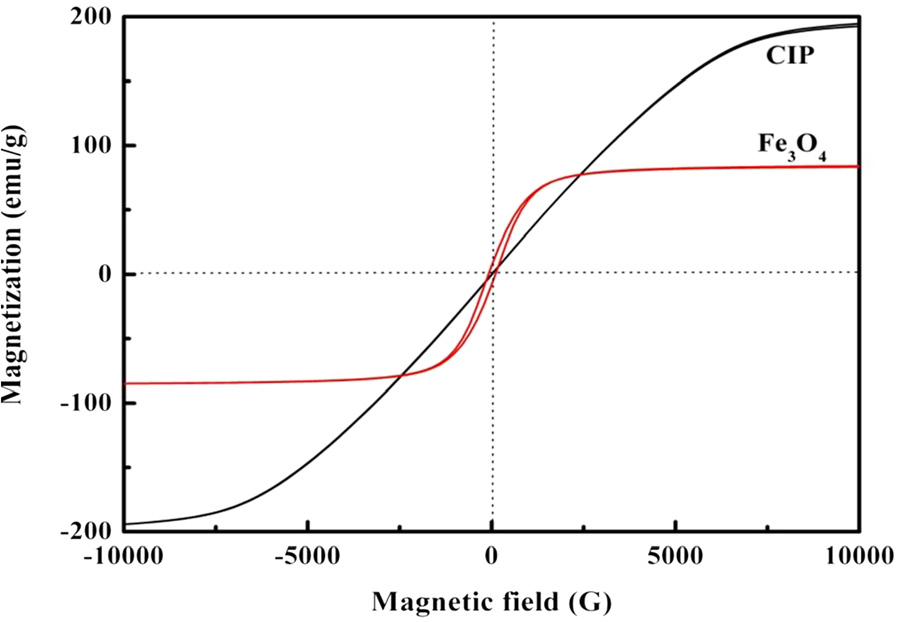
The hysteresis loops of Fe_3_O_4_ and CIP

The magnetization hysteresis loops of Fe_3_O_4_ particles and CIPs are shown in Fig. 3. It can be inferred from the hysteresis loop that Fe_3_O_4_ particles are typical soft magnetic materials. We can see that the *M*_*s*_ of Fe_3_O_4_ is 84.06 emu/g and the value of CIPs is 194.61 emu/g. It is reported that the existence of non-collinear spin structure which originated from the pinning of surface spins would result in the reduction of magnetic moment. The low *M*_*s*_ means Fe_3_O_4_ will be promptly saturated under an external magnetic field and the corresponding shear force induced by the field would not be as large as that of CIP which has higher *M*_s_.

**Fig. 4:**
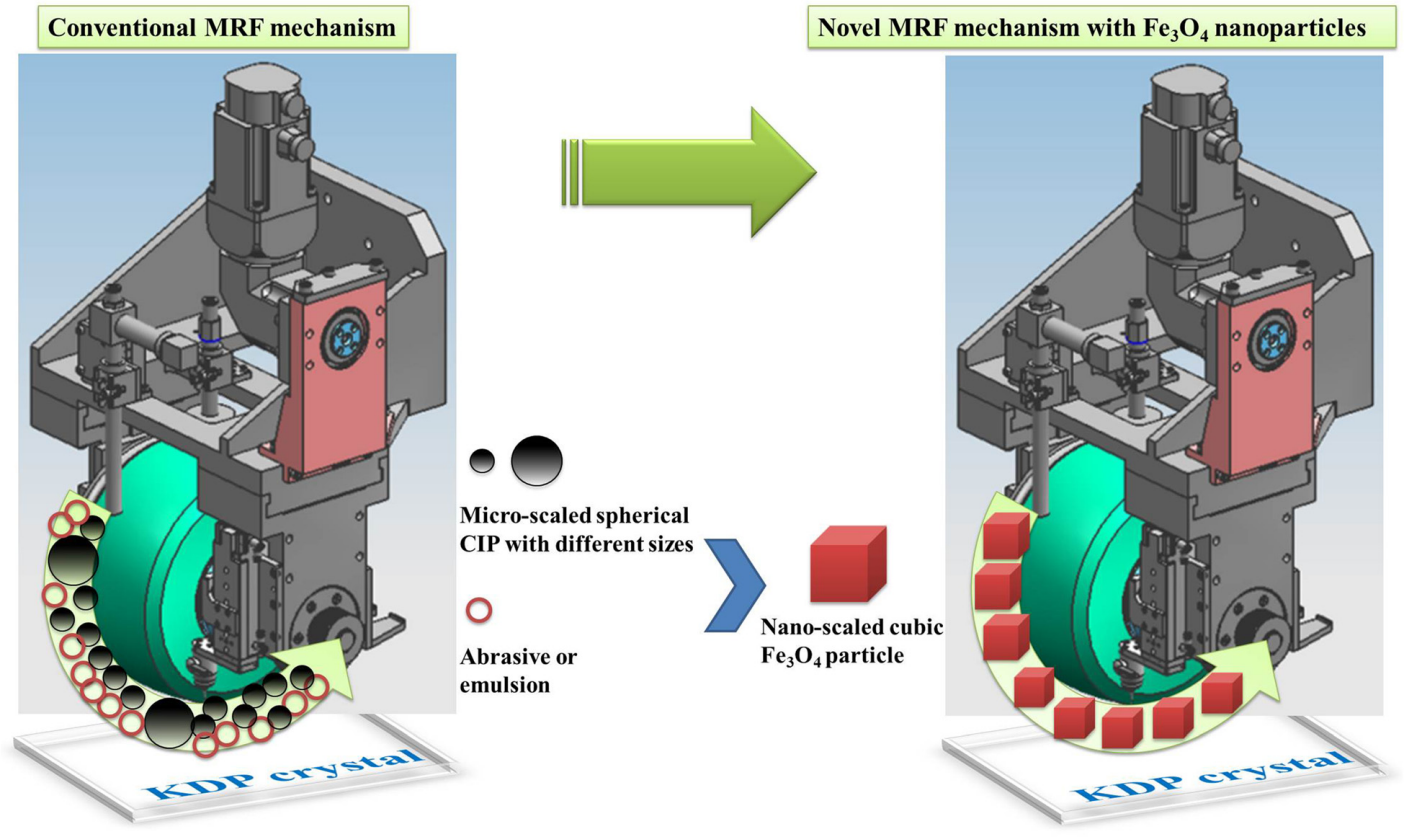
The sketch map of conventional CIP and abrasive which are substituted by Fe_3_O_4_ nanoparticles simultaneously

Based on the above analysis, we deduce that the small particle size and low shear stress may be beneficial to improve the surface quality in soft KDP finishing. Different from conventional magnetorheological finishing fluid that contains CIP and abrasive to generate a smooth surface, we design a novel finishing fluid by choosing the Fe_3_O_4_ nanoparticles to substitute both the CIP and abrasive as shown in Fig. 4.

**Fig. 5:**
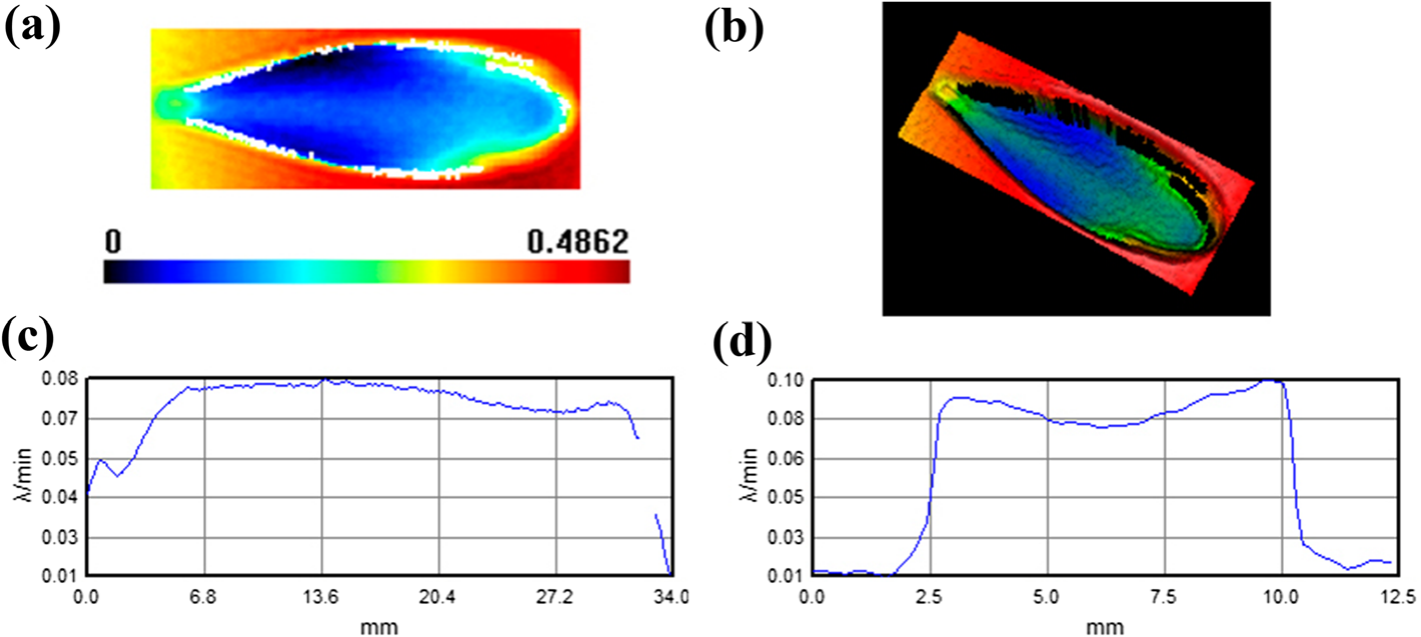
The 2D (**a**)/3D (**b**) morphologies and horizontal (**c**)/vertical (**d**) profiles of the finishing spot

The morphology and profile of finishing spot are exhibited in Fig. 5; the length is as large as 34 mm and the width is as large as 12.5 mm. The size is obviously bigger than conventional spots that are obtained on hard glasses with commercial aqueous CeO_2_/diamond finishing fluid containing CIPs [11]. However, the peak removal rate is only 0.097 λ/min and the volume removal rate is only 0.013 mm^3^/min, which are evidently lower than the value of commercial fluid between 4 and 30 λ/min [14]. This strange phenomenon can be easily understood by comparing the size and magnetic parameter of Fe_3_O_4_ and CIP.

Kordonski et al. have proposed the particle force model via taking the form of

1 $$ {G}_p=K\cdot \frac{\pi }{4}\cdot {\rho}_p\cdot {d}_p^4\cdot {\gamma}^2 $$

where *K* is the dimensionless coefficient, *ρ*_*p*_ is the density, *d*_*p*_ is the diameter, and *γ* is the shear rate [14]. Generally, the density of Fe_3_O_4_ (5.18 g/cm^3^) is obviously lower than that of CIP (7.845 g/cm^3^). Secondly, the diameter of nanosized Fe_3_O_4_ (100 nm) is much smaller than the microsized CIP (1 μm). So, according to Equation (1), the particle force of Fe_3_O_4_/CIP is 6.6 × 10^−5^ when at the same shear rate, indicating that the shear stress will be extremely weak compared with the value of CIP.

Furthermore, Miao et al. have calculated the shear stress by considering the magnetic field effectiveness via MRF experiments for a series of materials containing optical glasses and found a positive dependence of peak removal rate with shear stress [26]. The effect of both shear stress and mechanical properties are incorporated into a predictive equation for material removal as shown in

2 $$ {\mathrm{MRR}}_{\mathrm{MRF}}={C}_{P,\mathrm{M}\mathrm{R}\mathrm{F}\left(\tau,\ \mathrm{F}\mathrm{O}\mathrm{M}\right)}^{\mathit{\hbox{'}}}\frac{E}{K_c{H}_V^2}\cdot \tau \cdot V $$

where MRR is the material removal rate, ${C}_{P,\mathrm{M}\mathrm{R}\mathrm{F}\left(\tau,\ \mathrm{F}\mathrm{O}\mathrm{M}\right)}^{\mathit{\hbox{'}}}$ is a modification of Preston’s coefficient in terms of shear stress, *τ*. And $\frac{E}{K_c{H}_V^2}$ is the figure of material merit (FOM), where *E* denotes to Young’s modulus (resistance to elastic deformation), *H*_*V*_ is related to Vickers hardness (resistance to plastic deformation), and *K*_*c*_ is fracture toughness (resistance to fracture/crack growth). The *M*_*s*_ of Fe_3_O_4_ is much lower than that of CIP as shown in Fig. 3, hinting that the induced magnetic flux is not large enough and the corresponding shear stress that is controlled by the magnetic field is weak. The weak shear stress would inevitably result in low removal rate according to Equation (2). However, it does not mean that the lower the removal rate, the better the finishing fluid. Both smoothness and manufacturing efficiency should be considered to achieve a balance. That is why we select the cubic Fe_3_O_4_ nanoparticles rather than the smaller or spherical ones for the sake of utilizing spiculate friction of horns to maintain relative mechanical removal ability. Otherwise, too low removal rate would result in unacceptable finishing period. It should be noted that the shuttle-like spot is evidently different from conventional spot with D-shaped framework [11, 14]. This difference may be also arising from the small *M*_*s*_ of Fe_3_O_4_. The controllability of Fe_3_O_4_ under an external magnetic field is weak compared with CIP, especially in the area that is far from the center of the spot. So, the Fe_3_O_4_ nanoparticles in the fringe of the spot are loose and the spot is enlarged.

**Fig. 6:**
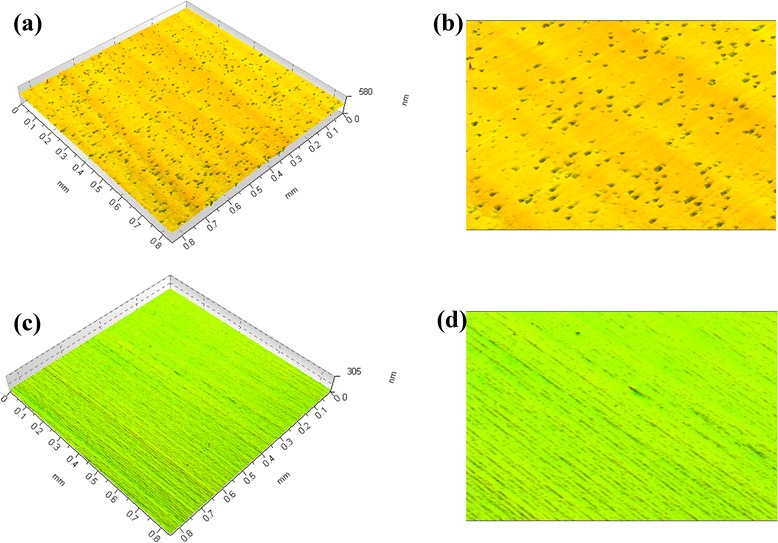
The 3D surface texture of the finished KDP with the fluid containing CIP (**a**) and Fe_3_O_4_ (**c**). **b** and **d** are the corresponding magnifications, respectively

Through the above discussion, it is easy to understand why the removal rate of the fluid containing Fe_3_O_4_ nanoparticles with much bigger and deeper spot is inversely lower than the value of conventional finishing fluid containing CIPs and CeO_2_/diamond abrasives. In order to validate our assumption that low removal rate may be in favor of the improvement of surface quality, we explore the surface texture of the finished KDP as shown in Fig. 6. Figure 6a, b clearly exhibits a great deal of “falling star” like pits and scratches; we may deduce these flaws are arising from the strike of CIP because the widths are micrometer-sized and just located in the CIP range. However, the surface texture is obviously improved if Fe_3_O_4_ is introduced as shown in Fig. 6c, d, few pits and scratches can be found, and there are only fine grooves which may be produced by the rolling and shaving actions between nanoparticles and KDP during finishing.

**Fig. 7:**
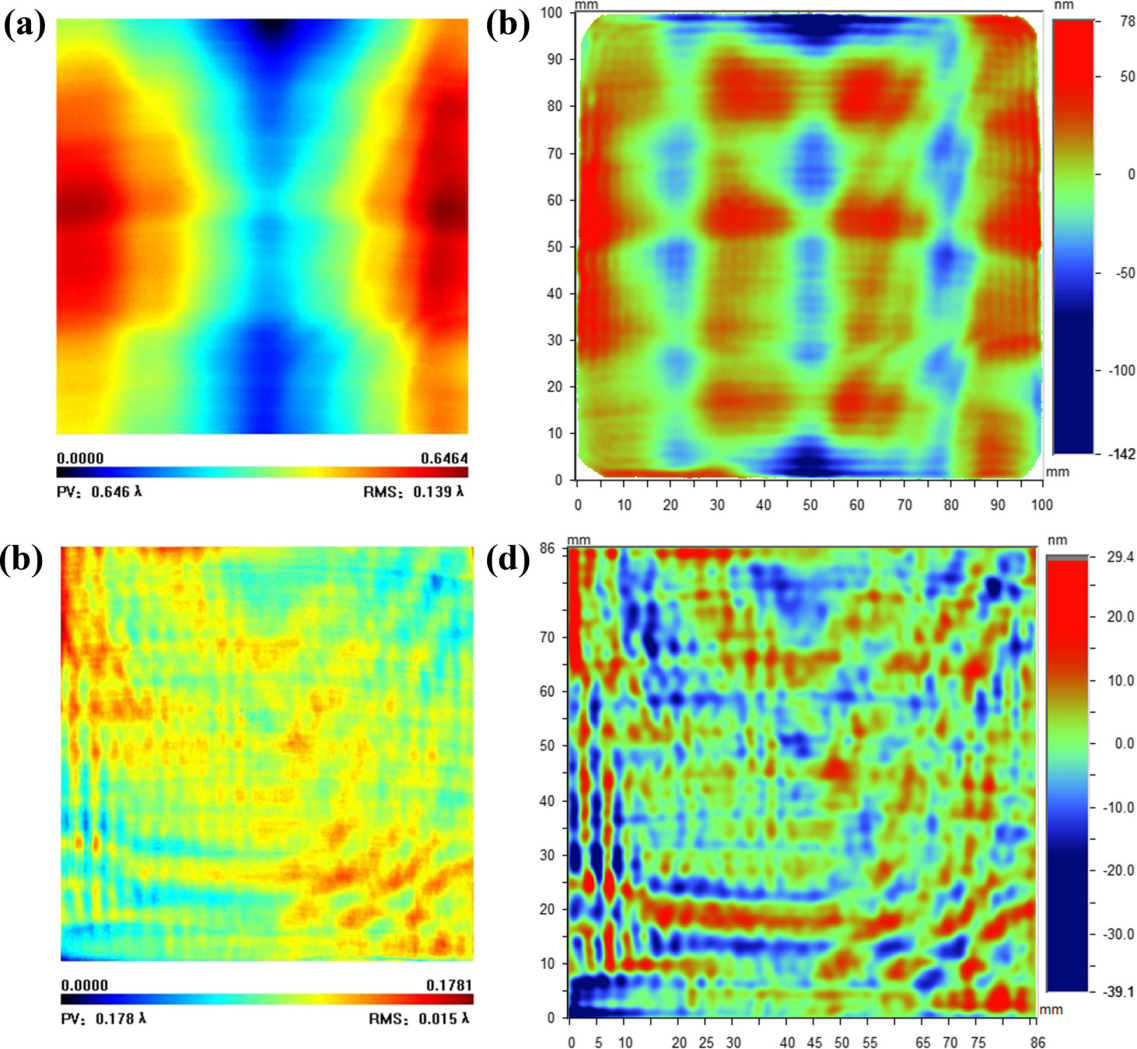
The KDP figure accuracy of PV and PSD1 before (**a**, **b**) and after (**c**, **d**) finishing

With the spot and removal function as discussed above, we take an experiment on a 100 mm × 100 mm KDP to display the finishing effectiveness of Fe_3_O_4_ nanoparticles instead of conventional CIPs and abrasives. The initial low-frequency figure accuracy PV is 0.646 λ (1 λ = 632 nm) as shown in Fig. 7a, and it is converged to 0.178 λ after MRF (Fig. 7c). In addition, the RMS of middle-frequency PSD1 is reduced from 23.083 to 6.539 nm as shown in Fig. 7b, d after MRF. All the data are characterized; waiting for 48 h after processing for the sake of releasing residual stress due to KDP is extremely sensitive to temperature variation and applied force. These inspiring results clearly prove the feasibility of utilizing Fe_3_O_4_ nanoparticles as a magnetic sensitive medium and an abrasive simultaneously. Continuous exploration to further improve surface quality on large-aperture KDP is under work.

## Conclusions

The size distribution of cubic Fe_3_O_4_ nanoparticles with sharp edges and horns is determined by TEM within 100–200 nm. A novel magnetorheological finishing fluid is designed by substituting conventional CIP and abrasive simultaneously with Fe_3_O_4_. The removal rate is extremely low compared with the value of conventional fluid though the finishing spot is much bigger, and the shuttle-like spot is different from the conventional D-shaped configuration. These strange phenomena are arising from the small size and inherently weak *M*_*s*_ of Fe_3_O_4_ by theoretical analysis. The finishing experiment exhibits nice surface texture and obvious convergence of figure accuracy PV and PSD1, validating the feasibility of our novel Fe_3_O_4_ design in MRF.
